# Model-free control for autonomous prevention of adverse events in robotics

**DOI:** 10.3389/frobt.2023.1271748

**Published:** 2024-01-05

**Authors:** Meenakshi Narayan, Ann Majewicz Fey

**Affiliations:** ^1^ Robotics and Automation Lab, Department of Engineering Technology, Miami University, Middletown, OH, United States; ^2^ Human-Enabled Robotic Technology Lab, Department of Mechanical Engineering, University of Texas at Austin, Austin, TX, United States

**Keywords:** model-free adaptive control, prevention of adverse events, minimally invasive surgery, autonomous robots, robotic needle steering

## Abstract

**Introduction:** Preventive control is a critical feature in autonomous technology to ensure safe system operations. One application where safety is most important is robot-assisted needle interventions. During incisions into a tissue, adverse events such as mechanical buckling of the needle shaft and tissue displacements can occur on encounter with stiff membranes causing potential damage to the organ.

**Methods:** To prevent these events before they occur, we propose a new control subroutine that autonomously chooses a) a reactive mechanism to stop the insertion procedure when a needle buckling or a severe tissue displacement event is predicted and b) an adaptive mechanism to continue the insertion procedure through needle steering control when a mild tissue displacement is detected. The subroutine is developed using a model-free control technique due to the nonlinearities of the unknown needle-tissue dynamics. First, an improved version of the model-free adaptive control (IMFAC) is developed by computing a fast time-varying partial pseudo derivative analytically from the dynamic linearization equation to enhance output convergence and robustness against external disturbances.

**Results and Discussion:** Comparing IMFAC and MFAC algorithms on simulated nonlinear systems in MATLAB, IMFAC shows 20% faster output convergence against arbitrary disturbances. Next, IMFAC is integrated with event prediction algorithms from prior work to prevent adverse events during needle insertions in real time. Needle insertions in gelatin tissues with known environments show successful prevention of needle buckling and tissue displacement events. Needle insertions in biological tissues with unknown environments are performed using live fluoroscopic imaging as ground truth to verify timely prevention of adverse events. Finally, statistical ANOVA analysis on all insertion data shows the robustness of the prevention algorithm to various needles and tissue environments. Overall, the success rate of preventing adverse events in needle insertions through adaptive and reactive control was 95%, which is important toward achieving safety in robotic needle interventions.

## 1 Introduction

Autonomous robots can perform complex tasks without human intervention using sensing, estimation, and control methodologies with the goal of improving the quality, efficiency, and safety of a system. These systems have vast applications in healthcare, commercial industries, transportation, aerospace, and defense. However, achieving human-like capabilities of dealing with high unpredictability, rapidly changing environments, and predicting adverse scenarios in a timely and safe fashion has been somewhat elusive for fully autonomous systems. Therefore, developing a combined approach of predictive control with adverse event prevention models, adaptive learning, decision making, and human–robot interaction techniques is an important step toward achieving smart and safe autonomy in various sectors of manufacturing, aerospace, and healthcare (Schwarting et al., 2018; Reis et al., 2021; Walter et al., 2022).

**FIGURE 1:**
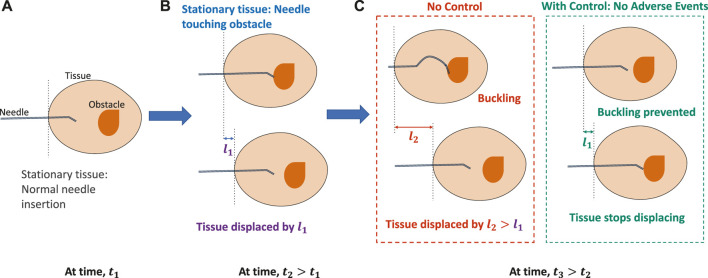
Importance of control for timely prevention of adverse events in robotic needle interventions. **(A)** When the tissue is stationary and the needle is inserted at time, *t*_1_. **(B)** On further insertion, when the needle encounters an obstacle or the tissue starts displacing by *l*_1_ at time, *t*_2_ > *t*_1_. **(C)** With no control, the needle either buckles or the tissue displaces further by *l*_2_ > *l*_1_, causing severe displacements of the tissue. However, with preventive control, needle buckling or tissue displacement is prevented.

An application area where safety is of utmost importance during autonomous behavior is in the field of robot-assisted minimally invasive surgery (RMIS). In RMIS, incision tools are maneuvered by a robot to assist surgeons in performing surgical operations more accurately while maintaining smaller incision cuts and improving patient outcomes (Poon et al., 2018; Khandalavala et al., 2019). Despite many research efforts to develop robotic systems for medicine, such as the da Vinci (Intuitive Surgical Inc., Sunnyvale, CA), Senhance (TransEnterix, Morrisville, NC, United States), and Flex^®^ (Medrobotics Corp., Raynham, MA, United States) robotic systems, an effective commercial system for deep-tissue needle insertion has not yet been realized due to complex needle–tissue interactions and the frequency of adverse events (Misra et al., 2010a; Majewicz et al., 2012). One such event is the buckling of a needle shaft that occurs due to rapid increase in frictional forces when the needle encounters a hard membrane or an obstacle (Narayan et al., 2018a). On further insertion, buckling could lead to tissue ruptures (Gruijthuijsen et al., 2018). Another event that could prevent surgeons from reaching desired targets within the tissue is the tissue deformation and displacements caused by the insertion forces of the needle (Oldfield et al., 2015). To address these difficulties, several steering control methods and novel needle designs have been proposed that accurately control the needle tip to reach desired targets (Hadjerci et al., 2016; Sprang et al., 2016; Li et al., 2017b; Fallahi et al., 2017). There have been efforts to autonomously predict adverse situations and correct for faults using sensor readings in unmanned vehicles (Huang et al., 2022; Ryu et al., 2022); however, much work is still needed for safe autonomous predictions of adverse events in robotic surgery. While reducing targeting errors is almost a solved problem, preventing and controlling adverse events such as needle buckling and tissue displacements before occurrence still remain a significant challenge to be addressed in real time (Leibinger et al., 2016; de Baere et al., 2022). With appropriate prediction and control subroutines, these adverse events can be prevented before they occur and damage to the tissue could be reduced, as demonstrated in Figure 1.

In prior work, we developed novel sensor forecasting algorithms based on which needle buckling events were predicted before occurrence (Narayan and Fey, 2020). Furthermore, with unknown needle–tissue interaction dynamics and computation complexities, implementing model-based or learning-based controllers would be challenging (Li et al., 2017a). Therefore, we implement a model-free adaptive control (MFAC) technique (Hou and Jin, 2011) for steering control of needles and minimizing adverse events. This technique relies on the principle of linearizing a nonlinear system with a single parameter called the pseudo partial derivative (PPD) parameter which is *slowly* time varying in nature. However, this technique is not robust to changes in system parameters which affect real-time stability and output convergence. Two key contributions of this paper are: 1) developing an improved version of MFAC called improved model-free adaptive control (IMFAC) for general nonlinear systems by introducing *fast* time-varying PPD to guarantee continuous real-time stability, output convergence, and robustness against any external disturbance and 2) developing an action subroutine that integrates IMFAC with prior adverse event prediction methods (Narayan and Fey, 2020) to autonomously prevent needle buckling and tissue displacement events during robotic needle interventions.

This paper is structured as follows: Section 2 discusses the literature for adverse event prevention methods and data-driven control models related to robot-assisted needle insertions. Section 3 discusses the theoretical development of IMFAC, stability analysis, and simulation tests on a general class of nonlinear systems. Section 4 summarizes control algorithms to prevent adverse events. Section 5 discusses experimental methods. Section 6 discusses adverse event prevention results during needle insertions in gelatin and biological tissues. Finally, Section 7 concludes the paper.

## 2 Related work

This section discusses existing methods to prevent adverse events specific to robotic needle interventions, as well as state-of-the-art data-driven control methods.

### 2.1 Prevention and control of adverse events

Over the years, several strategies have been implemented to avoid adverse events within the tissue, such as needle buckling and target displacements (Rossa and Tavakoli, 2017). For example, to prevent needle buckling events as a result of increases in the needle–tissue interaction forces, novel designs for steerable needles were developed. The designs increase the critical buckling force of the needle shaft and eventually reduce insertion forces on the needle during insertions (Webster et al., 2006; Van De Berg et al., 2017). However, these methods do not provide real-time buckling control. It is more important to track the difference between insertion force and critical buckling force relative to the needle position in the tissue, rather than just the critical buckling force (Van De Berg et al., 2017; Hulburt et al., 2019). For this purpose, needle–tissue biomechanics models are used to derive a relationship between insertion forces and needle displacements (Tang et al., 2007; Misra et al., 2010b; Sakes et al., 2016). However, biomechanics models require prior information of tissue properties, which is challenging for intra-operative procedures when tissue environments are unknown. Similarly, imaging techniques (Abolhassani et al., 2006; Hadjerci et al., 2016; Li et al., 2017b), mechanical devices and probes as tissue constraints (Brown et al., 2010; Kobayashi et al., 2012), and path planners (Vrooijink and Abayazid, 2014; Li et al., 2017b; de Baere et al., 2022) have been implemented to control the deformations and displacements of the tissue. Though these methods have shown success in achieving accurate target placement of the needle tip, they rely on finite element and biomehanics models. Accurate characterization of needle–tissue dynamics can be time consuming, and thus, it can be challenging to implement real-time control (Rossa and Tavakoli, 2017; Barnoy et al., 2022).

Recently, research groups have taken inspiration from parasitic invertebrates such as wasp ovipositor and mosquito to design needles that can penetrate solid substrates easily. Inspired by the mechanism of the ovipositor, a thin needle composed of several wires sliding alongside the other was developed to maneuver needles with high curvatures while minimizing impact on the surrounding tissues (Sprang et al., 2016; Scali et al., 2017). Similarly, Li et al. (2020) developed a needle-cannula system that mimics the mosquito proboscis mechanism of incremental motions to reduce tissue deformations and displacements during insertion. They also used harpoon notches at the needle tip to reduce needle–tissue frictional forces (Li et al., 2020). These bio-inspired mechanisms of needle insertions can generally improve maneuverability, while reducing critical needle–tissue interaction events such as needle buckling, tissue deformations, target displacement, and needle deflections. However, they have complicated designs and require several fixtures to prevent buckling of individual wires. Furthermore, a systematic study to select design parameters is required, which usually change due to varying heterogeneous tissue substrate (Leibinger et al., 2016; Matheson and Rodriguez y Baena, 2020).

These limitations call for alternative solutions that do not depend on needle designs and unknown tissue structures, such as model-free techniques. Thus, optimizing on needle insertions while minimizing adverse events in real time using data-driven control techniques would be the focus of this work.

### 2.2 Data-driven control methods

In the last decade, much attention has been given to the application of data-driven control techniques for modeling complex nonlinear processes (Abouaïssa and Chouraqui, 2019). The common classical technique being the proportional, integral, and derivative (PID) controller has shown to be generalizable to many systems and finds applications in most regulatory-based process control industries (Åström and Hägglund, 1995; McMillan, 2012). However, tuning of the gains is performed offline and is affected by process disturbances and the time-varying behavior of the system. This makes PID less robust and challenging to implement in real time. To address these issues, intelligent PID was proposed to predict dynamic system behavior through estimation techniques and online numerical differentiator, eliminating offline tuning and prior identification procedure (Fliess and Join, 2009). Yet, its performance is limited by issues of sensor noise, sampling rates, and computation power as the size of data increases (Hou and Jin, 2011).

There has been increasing interest in unconventional control strategies such as neural networks, fuzzy logic, and genetic algorithms (Thomas and Poongodi, 2009; Heidari et al., 2013; He et al., 2015; Bing et al., 2018; Glida et al., 2021). Neural network control is based on learning from the mapping between system input–output data, whereas fuzzy control is based on learning from past experience and expert knowledge to predict and control system behavior (Abouaïssa and Chouraqui, 2019). Genetic algorithms tune control models based on stochastic optimization techniques that mimic biological evolution (Rodríguez-Abreo et al., 2020). The advantage is that these methods do not require any mathematical model of the system; however, determining parameters that accurately satisfy system behavior can be computationally expensive. Moreover, methods to identify system stability with learning models are still yet to be developed and fully analyzed (Singh and Sukavanam, 2012). Efforts have been made to develop learning-based control techniques with low computation complexity, to optimize needle insertions in tissue while minimizing undesired needle deflections and tissue displacements (Buzurovic et al., 2010; Rossa et al., 2017; Pratt and Petruska, 2022). Yet, their methods do not entail prevention of these events before occurrence.

An alternative technique to control any complex nonlinear system without using learning models is MFAC (Hou and Jin, 2011). This technique uses the dynamic linearization principle to simplify the system nonlinearities and requires only system input–output data. Due to the ability to perform stability analysis online, simplified mathematical models, and robustness against external disturbances, MFAC finds applications in unmanned aerial vehicles, automated manufacturing, and mobile robots (Hou and Jin, 2019). Recently, Li et al. (2017a) derived a model-free adaptive control from Kalman filter techniques to predict needle buckling and control positions of a flexible robot. However, they have not provided a mechanism yet to minimize an adverse event when it occurs. To the best of our knowledge, model-free adaptive control has not yet been implemented on robotic systems that could control needle–tissue interactions while preventing or minimizing adverse events in real time.

## 3 Improved model-free adaptive control

In the original MFAC framework, the online stability of a system depends on the choice of system and control parameters (Hou and Jin, 2011). Since these parameters are tuned offline, real-time robustness against system uncertainties and output convergence cannot be guaranteed. Therefore, online tuning of control parameters is implemented with MFAC to improve real-time output convergence and stability in the presence of unexpected disturbances, and we name this technique as IMFAC. This section focuses on the formulation of IMFAC and parametric selection methods through theoretical analysis of stability and output convergence.

### 3.1 Derivation

If a discrete-time nonlinear system with output *y* and input *u*_*c*_ satisfies the *Lipschitz* condition

$$\left|y\left(k_{i}\right)-y\left(k_{j}\right)|\leq \tilde{b}|u_{c}\left(k_{i}\right)-u_{c}\left(k_{j}\right)\right|$$ (1)

for timestamps *k*_*i*_ ≠ *k*_*j*_ and *i*, *j* > 0, then the nonlinear system can be approximated as a dynamic linearization model,

$$y\left(k+1\right)=y\left(k\right)+\Theta_{c}\left(k\right)\Delta u_{c}\left(k\right),$$ (2)

where Θ_*c*_ is a slow time-varying parameter called PPD that compresses system nonlinearity. This approximation is useful in predicting the output of a nonlinear system without using dynamic models. PPD is a scalar for single-input single-output (SISO) systems and a vector for multi-input multi-output (MIMO) systems. For tracking and regulation-based control, the following quadratic cost function is employed to obtain control effort, Δ*u*_*c*_(*k*), as a function of prediction errors, y* − *y*(*k* + 1):

$$J_{c}\left(u_{c}\left(k\right)\right)={\left|y^{*}-y\left(k+1\right)\right|}^{2}+\lambda_{c}\left|u_{c}\left(k\right)-u_{c}\left(k-1\right)\right|,$$ (3)

where y* is the desired output of the system. The weighting factor *λ*_*c*_ > 0 restricts Δ*u*_*c*_(*k*) and, thus, is an important tuning parameter for output convergence and system stability. Substituting *y*(*k* + 1) from Eq. 2 and minimizing Eq. 3 with respect to *u*_*c*_, the optimal input for tracking is computed as

$$u_{c}\left(k\right)=u_{c}\left(k-1\right)+\frac{\Theta_{c}\left(k\right)}{\lambda_{c}+\Theta_{c}\left(k\right).\Theta_{c}{\left(k\right)}^{T}}\left(y^{*}-y\left(k\right)\right),$$ (4)

where Θ_*c*_(*k*) is determined using numerical approximation methods due to unknown system dynamics. The following cost function is used to obtain an approximate Θ_*c*_(*k*) by minimizing the error between the modeled output from Eq. 2 and observed output, *y*(*k*):

$$\begin{array}{c} J_{c}\left(\Theta_{c}\left(k\right)\right)={\left|y\left(k\right)-y\left(k-1\right)-\Theta_{c}\left(k\right)\Delta u_{c}\left(k-1\right)\right|}^{2}+{\left|\Theta_{c}\left(k\right)-\Theta \left(k-1\right)\right|}^{2} \end{array}.$$ (5)

The second term in the cost function ensures robustness against system nonlinearity. Minimizing this cost with respect to Θ_*c*_(*k*) yields

$$\Delta \Theta_{c}\left(k\right)=\frac{\Delta u_{c}{\left(k-1\right)}^{T}.\left(\Delta y\left(k\right)-\Theta_{c}\left(k-1\right).\Delta u_{c}\left(k-1\right)\right)}{1+\Delta u_{c}{\left(k-1\right)}^{T}.\Delta u_{c}\left(k-1\right)},$$ (6)

where Δ*u*_*c*_(*k*) = *u*_*c*_(*k*) − *u*_*c*_(*k* − 1). However, Eq. 6 is not robust to process errors such as arbitrary initialization of system parameters, which affect output convergence. This issue is resolved when Θ_*c*_(*k*) is assigned as a *rapid* time-varying parameter

$$\begin{array}{cc} \text{If }\;|\Delta y\left(k+1\right)| & >\tilde{b}|\Delta u_{c}\left(k\right)|\\ \Theta_{c}\left(k\right) & =\frac{\left|\Delta u_{c}\left(k\right)\right|}{\left|\Delta y\left(k+1\right)\right|} \end{array}.$$ (7)

The *Lipschitz* constant, $\tilde{b}$ , is chosen from the theoretical analysis of system stability and output convergence. Finally, Eqs 2–7 are computed iteratively. This technique is similar to the existing framework (Hou and Jin, 2011); however, the novelty lies in the introduction of the *rapid* varying PPD and the methods of initializing control parameters, (*λ*_*c*_, Θ_*c*_(0)), and system parameters, (*u*_*c*_(0), *y*(0)), for continuous real-time stability discussed in Section 3.2.

### 3.2 Stability analysis and parameter initialization

In the traditional MFAC scheme, Θ_*c*_(0), *y*(0), *u*_*c*_(0), and *λ*_*c*_ are hand-tuned using trial and error methods which can be tedious. This issue can be resolved if a systematic approach to parametric initialization is followed under certain assumptions of the system stated below.

Assumption 1 *The nonlinear system* (1) *satisfies a special case of Lipschitz condition for all* *k* > 0:

$$\left|y\left(k+1\right)-y\left(k\right)\right|\leq \tilde{b}\left|u_{c}\left(k\right)-u_{c}\left(k-1\right)\right|,$$ (8)

*where* *u*_*c*_(*k*) ≠ *u*_*c*_(*k* − 1) *and* $\tilde{b}\in \left(0,1\right)$ *. Therefore,* |Θ_*c*_| ∈ (0, 1).

This assumption holds for a class of systems that cannot move beyond the commanded external force, unless affected by disturbance factors. For example, in robotic needle steering, a tissue cannot displace beyond the needle insertion velocity unless affected by other factors such as patient breathing. This condition resembles the bounded-input bounded-output stability where energy in the system output cannot increase beyond the energy levels in the system inputs according to energy conservation law. Hence, the absolute values of Θ_*c*_ from Eq. 2 are also bounded between 0 and 1 according to Eq. 8.

Next, the sign of Θ_*c*_ depends on the system response to inputs, which is stated in the following assumption.

Assumption 2 *If system output changes in the same direction as the inputs, then* Θ_*c*_ ∈ (0, 1)*. Otherwise,* Θ_*c*_ ∈ (−1, 0).

This assumption holds for systems whose outputs are affected by multiple inputs. For example, if two inputs (say, *u*_*c*1_ and *u*_*c*2_) influence an output such that an increase in *u*_*c*1_ and decrease in *u*_*c*2_ drive the system to desired outputs, then 0 < Θ_*c*_(0) < 1 for *u*_*c*1_ and −1 < Θ_*c*_(0) < 0 for *u*_*c*2_.

Third, if initial values of the system input and output are assigned close to the operating points, then output convergence is guaranteed.

Assumption 3 *Output convergence,* $\underset{k\to \infty }{\lim}|y\left(k+1\right)|\to y^{*}$ , *is guaranteed if* −*a* < *y*(0) < *a* *and* $\tilde{a}<u_{c}\left(0\right)<\tilde{a}$ *such that* |y* − *y*(0)| → 0 *for some* *a* > 0*,* $\tilde{a}>0$ .

The operating points $\left(a,\tilde{a}\right)$ are usually identified during prior simulations. For physical systems, they are easily identified as safe operating limits provided by the system manufacturer.

Finally, output convergence also depends on the range of the input weighting factor, *λ*_*c*_, stated as follows.

Assumption 4 *If system* (1) *is controlled for a regulation problem, then there exists a minimum value,* $\lambda_{c}^{\min}=\frac{1}{4}$ *such that* |y* − *y*(*k* + 1)| → 0 *for all* *k* *and* $\lambda_{c}>\lambda_{c}^{\min}$ *. There also exists a maximum value,* $\lambda_{c}^{\max}$ , *above which the system settles but with steady-state errors.*

Proof. The proof is straightforward where the boundedness property of $\tilde{b}$ from Assumption 1 is used to maintain both system stability and convergence. If the steady-state error is defined as

$$e\left(k+1\right)=y^{*}-y\left(k+1\right),$$ (9)

then substituting *y*(*k* + 1) in Eq. 9 from Eq. 1, we have

$$e\left(k+1\right)=y^{*}-y\left(k\right)-\tilde{b}\Delta u_{c}\left(k\right).$$ (10)

Substituting Δ*u*_*c*_(*k*) from Eq. 4 in Eq. 10 and from the absolute value theorem,

$$|e\left(k+1\right)|\leq \left|1-\frac{\tilde{b}\Theta_{c}^{T}}{\lambda_{c}+\Theta_{c}\Theta_{c}^{T}}\right||e\left(k\right)|.$$ (11)

Now, using the boundedness of Θ_*c*_ from Assumption 2, we know that

$$0<|1-\frac{\tilde{b}\Theta_{c}^{T}}{\lambda_{c}+\Theta_{c}\Theta_{c}^{T}}|<1.$$ (12)

Since $\tilde{b}$ cannot exceed 1 from Eqs 11, 12, we assume $|\frac{1.\Theta_{c}^{T}}{\lambda_{c}+\Theta_{c}\Theta_{c}^{T}}|<1$ as the maximum limit to find $\lambda_{c}^{\min}$ . Then, using the general concept of quadratic equation, ${\left(\sqrt{\lambda_{c}}\right)}^{2}+\Theta_{c}\Theta_{c}^{T}\geq 2\left(\sqrt{\lambda_{c}}\right)\Theta_{c}$ , we have

$$|e\left(k+1\right)|\leq \left|1-\frac{\Theta_{c}^{T}}{\lambda_{c}+\Theta_{c}\Theta_{c}^{T}}\right||e\left(k\right)|\leq \left|1-\frac{1}{2\sqrt{\lambda_{c}}}\right|<1.$$ (13)

Hence, $\lambda_{c}^{\min}=0.25$ using the relation, $\frac{1}{2\sqrt{\lambda_{c}}}=1$ in Eq. 13. Finding an exact maximum value, $\lambda_{c}^{\max}$ , is not important, as *λ*_*c*_ > 1 leads to lower rising times and larger steady-state errors for many systems (Hou and Jin, 2011). Since many real-time applications require fast rising time and low steady-state errors, $\lambda_{c}^{\max}=1$ is considered. Furthermore, Δ*u*_*c*_(*k*) needs to be constrained as most actuators have finite excitation. Thus, *λ*_*c*_ can be chosen arbitrarily in the range $\left(\lambda_{c}^{\min},1\right)$ to balance between constrained inputs and tracking errors.

### 3.3 Simulation tests

The improved framework (IMFAC) is validated on two test nonlinear systems to compare performance with MFAC (Hou and Jin, 2011). All equations are coded in the MATLAB^®^ 2018Rb environment and tested on simulated SISO and MIMO nonlinear systems. The algorithms are compared using the same initial conditions and control parameters.

For a SISO system with some nonlinear input–output model,

$$\begin{array}{ll} & y\left(k+1\right)\\ & =\left\{\begin{array}{c} \frac{y\left(k\right)}{1+y{\left(k\right)}^{2}}+u_{c}^{3}\left(k\right);\;k\leq 500\;\\ \frac{y\left(k\right)y\left(k-1\right)y\left(k-2\right)u_{c}\left(k-1\right)\left(y\left(k-2\right)-1\right)+\beta \left(k\right)u_{c}\left(k\right)}{1+y{\left(k-1\right)}^{2}+y{\left(k-2\right)}^{2}};\;k>500,\beta \left(k\right)=\text{round}\left(k/500\right)\; \end{array}\right.. \end{array}$$ (14)

**FIGURE 2:**
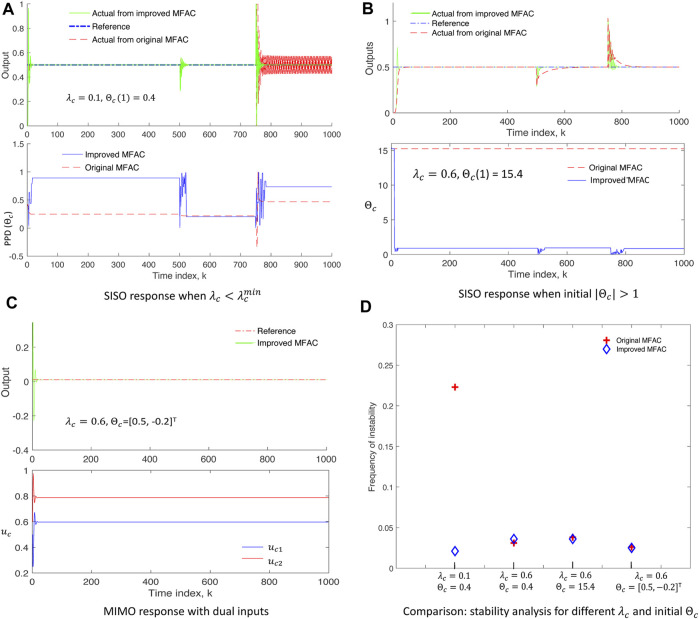
Simulation results on test nonlinear systems: **(A,B)** SISO system showing stable response and faster convergence of improved MFAC (solid green, upper plots) even when $\lambda_{c}<\lambda_{c}^{\min}$ or |Θ_*c*_| > 1, due to the *rapid* time-varying behavior of Θ_*c*_ (solid blue, lower plots). **(C)** MIMO system response through the directional control of inputs (*u*_*c*1_, *u*_*c*2_) and initial Θ_*c*_, where the output is controlled by increasing *u*_*c*1_ with positive Θ_*c*_ and decreasing *u*_*c*2_ with negative Θ_*c*_. **(D)** Stability analysis comparing improved MFAC (blue diamond) and original MFAC (red cross) for different *λ*_*c*_ and initial Θ_*c*_.

Let the initial conditions of the system be *u*_*c*_(1) = *u*_*c*_(2) = 0 and *y*(1) = *y*(2) = 0 with a constant reference output as y* = 0.5. The output of the system is expected to change at *k* = 500, 750 according to Eq. 14, and thus, some disturbances due to these transitions are expected. Simulation results of SISO are shown in Figures 2A, B, where solid green plots correspond to IMFAC, dashed red plots correspond to original MFAC, and the dash–dot blue plot is the reference signal. For $\lambda_{c}=0.1<\lambda_{c}^{\min}$ , IMFAC converged to the desired value within *k* = 790 after disturbance at *k* = 750, while MFAC showed lack of output convergence due to sustained oscillations (upper plot of Figure 2A). When |Θ_*c*_| > 1, IMFAC showed faster convergence at the disturbances, whereas MFAC showed slower convergence at *k* = 830 after disturbance at *k* = 750 (upper plot of Figure 2B). For the same control parameters and initial conditions, IMFAC showed better stability and faster convergence due to the corrective action of the *rapid* time-varying PPD, compared to MFAC with *slow* time-varying PPD (lower plots of Figures 2A, B).

We consider a two-input (*u*_*c*1_, *u*_*c*2_) one-output (*y*) MIMO system with some nonlinear model in Eq. 15.

$$y\left(k+1\right)=\frac{5y\left(k\right)+2u_{c1}\left(k\right)-3u_{c2}{\left(k\right)}^{2}+2u_{c1}{\left(k\right)}^{2}}{5+u_{c1}\left(k\right)+5u_{c2}\left(k\right)},$$ (15)

where $u_{c}={\left[u_{c1},u_{c2}\right]}^{T}\in R^{2\times 1}$ . Arbitrary initial conditions of the system are *u*_*c*_(1) = *u*_*c*_(2) = [0.5,0.6]^*T*^, *y*(1) = 0, *y*(2) = 0.9, and Θ_*c*_(1) = [0.5, −0.2]. Let *λ*_*c*1_ = *λ*_*c*2_ = *λ*_*c*_ = 0.5 for each input. We can have *λ*_*c*1_ ≠ *λ*_*c*2_; however, for the ease of validating Assumption 2, we choose *λ*_*c*1_ = *λ*_*c*2_. A constant reference trajectory close to zero (y* = 0.01) is considered since ideal y* = 0 would cause control inputs to be zero which cannot drive the system to desired outputs. Therefore, *near* zero reference is used to simulate stationary or minimum movement of the system. Simulation results show that negative Θ_*c*2_ = −0.2 decreases *u*_*c*2_ and positive Θ_*c*1_ = 0.5 increases *u*_*c*1_, which drive the system to the desired output (y* = 0.01), see Figure 2C. It should be noted that initial Θ_*c*_ can be assigned to control the system inputs in the desired direction only if there is basic knowledge about system response to the inputs.

Finally, the performance of IMFAC is compared with original MFAC in terms of frequency of unstable events computed as the number of occurrences of |Δ*y*(*k* + 1)| > |Δ*u*_*c*_(*k*)| per simulation time, for different values of control parameters (*λ*_*c*_ and Θ_*c*_) as shown in Figure 2D. The blue diamond plot (IMFAC) for $\lambda =0.1<\lambda_{c}^{\min}$ shows that system stability was not affected by arbitrary initialization of *λ*_*c*_ and other disturbances. Otherwise, these results show the comparable performance of IMFAC with MFAC using a systematic approach to parameter initialization. On average, the convergence time of IMFAC was 20% faster than that of MFAC. This shows that irrespective of the control parameters and initial conditions, system stability and fast output convergence are always achieved with IMFAC.

### 3.4 Application to robotic needle steering

**FIGURE 3:**
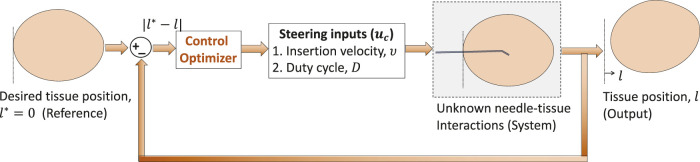
Model-free control to minimize tissue motion *l*.

The MIMO system in Section 3.3 is similar to the needle intervention applications where tissue motion, *y* = *l*, is controlled by two inputs: a) insertion velocity *ν* and b) duty-cycle factor as a measure of the number of needle shaft rotations per insertion, *D*. Tissue motion can be minimized with a decrease in insertion velocity and increase in the duty-cycle factor. Therefore, stationary tissue is desired (l* → 0.01) during needle insertion procedures. Through appropriate selection of parameters (*λ*_*c*_, Θ_*c*_), IMFAC can minimize tissue motions during needle insertions as shown in Figure 3. The input–output equations of the SISO and MIMO systems from Section 3.3 are used only for simulation purpose to validate the control algorithms and are not available in real time. For real-time needle insertion experiments in unknown tissues, *l* is obtained from position sensor measurements, and inputs are the user-defined steering control inputs, *ν* and *D*. Based on the previous MIMO simulation results, initial values of Θ_*c*_ = [0.5,−0.2]^*T*^ and same *λ*_*c*_ = 0.5 will be used for both the inputs (*ν* and *D*) for all needle insertions. Initial values of the inputs are commanded by a user. The range or operating points of these inputs is decided by the maximum rating of the motors used to drive the needle following Assumption 3. Since the output (tissue displacement) is zero when excitation (insertion inputs) is zero for needle–tissue systems, we will assume zero initial values, *l*(0) = 0, for all needle insertions. The next section will discuss application of IMFAC to prevent adverse events during needle insertions in a tissue.

## 4 Prevention of adverse events

In this section, we first summarize the methods to predict adverse events using a previously developed sensor data forecasting technique called compact-form dynamic linearization model-free prediction (CFDL-MFP) (Narayan and Fey, 2020). We then develop optimal needle steering algorithms using IMFAC and detection algorithms from the work of Narayan et al. (2018b) for the overall prevention of adverse events.

### 4.1 Prediction of adverse events from sensor data forecasts

Let the time series data obtained from a sensor at current time *k* be *S*(*k*). The steps to generate sensor data forecasts, ${\hat{S}}_{N}\left(k+1\right)$ , for some future prediction horizon, *N*, using CFDL-MFP are

$${\hat{S}}_{N}\left(k+1\right)={\hat{S}}_{N}\left(k\right)+{\hat{\Phi }}_{N}\Delta U_{N}\in R^{N}.$$ (16)

The data vector *S* represents sensor data. For example, previous *N* samples of force data measured from a force sensor at time instant *k* are represented as *S*_*N*_(*k*), and ${\hat{S}}_{N}\left(k+1\right)$ represents *N*-step ahead forecasts of force data at the next time instant *k* + 1. The forecast parameters Δ*U*_*N*_(*k*) and ${\hat{\Phi }}_{N}\left(k\right)$ are computed following the procedure in our previous work (Narayan and Fey, 2020):

$$\begin{array}{ll} {\hat{\Phi }}_{N}\left(k\right) & ={\left(\Delta U_{N}\left(k-1\right)+\vec{1}\right)}^{◦-1}◦\left({\hat{\Phi }}_{N}\left(k-1\right)+\Delta U_{N}\left(k-1\right)◦\Delta {\hat{S}}_{N}\left(k\right)\right)\\ \Delta U_{N}\left(k\right) & =\frac{{\hat{\Phi }}_{N}\left(k\right)}{\left(\lambda .\vec{1}+2{\hat{\Phi }}_{N}^{2}\left(k\right)\right)}◦\left(S_{N}^{*}\left(k\right)+S_{N}\left(k\right)-2{\hat{S}}_{N}\left(k\right)\right) \end{array},$$ (17)

where ◦ is array element-wise operation, $\vec{1}$ is an *N*-dimension vector of ones, and $\Delta \hat{S}\left(k\right)=\hat{S}\left(k\right)-\hat{S}\left(k-1\right)$ . The sensor data *S*_*N*_(*k*) contain previous *N* observed data points, and the reference vector $S_{N}^{*}\left(k\right)$ is the running average of the observed data points.

Forecasts of force sensor data are generated when a slight increase in force is detected, and if force forecasts show patterns of rapid increase, then needle buckling is predicted (Narayan and Fey, 2020). Similarly, tissue displacements can be predicted using forecasts of tissue position data. Tissue displacement is characterized based on the intensity of motion such as mild tissue displacement (MTD) and severe tissue displacement (STD). STD occurs when the needle tip stops moving relative to the tissue frame (Narayan et al., 2018a). To predict STD before occurrence, *N*-step forecasts of the tissue position relative to fixed frame ${\hat{l}}_{\mathrm{twN}}$ and the needle-tip position relative to fixed frame ${\hat{l}}_{\mathrm{nwN}}$ are generated using Eqs 16, 17. It should be noted that *l*_*tw*_ and *l*_*nw*_ are the Euclidean norm of 3D positions measured from position sensors ${\vec{p}}_{tw}$ and ${\vec{p}}_{nw}$ , respectively. The norm of the needle-tip position relative to the tissue frame is obtained as *l*_*nt*_ = *l*_*nw*_ − *l*_*tw*_, and $\left({\hat{l}}_{\mathrm{twN}},{\hat{l}}_{\mathrm{nwN}},{\hat{l}}_{\mathrm{ntN}}\right)$ are the forecasts of the corresponding position norms. STD events are then predicted using these norm position forecasts with the sigmoid slope detection methods in Eq. 18.

$$\begin{array}{cc} {\hat{m}}_{t}\left(k+i\right) & =\frac{180}{\pi }.\frac{\Delta {\hat{l}}_{N}\left(k+i\right)}{\Delta \hat{s}\left(k+i\right)};\;i=1,2,.,N;\;{\hat{l}}_{N}=\left({\hat{l}}_{\mathrm{twN}},{\hat{l}}_{\mathrm{nwN}},{\hat{l}}_{\mathrm{ntN}}\right)\\ \hat{g}\left({\hat{m}}_{t}\left(k+i\right)\right) & =\frac{1}{1+e^{-{\hat{m}}_{t}\left(k+i\right)}};\;\hat{g}=\left({\hat{g}}_{nw},{\hat{g}}_{tw},{\hat{g}}_{nt}\right) \end{array},$$ (18)

$$\begin{array}{ll} & \text{If }{\hat{g}}_{tw}\left(k+i\right)>m_{2}\text{ OR }{\hat{g}}_{tw}\left(k+i\right)<m_{1}\\ & \text{ Then }\left\{\begin{array}{c} \begin{array}{ccc} & \text{If }m_{1}<{\hat{g}}_{nt}\left(k+i\right)<m_{2};\; & \text{ STD predicted}\\ & \text{Else }\;\;\;\;\; & \text{MTD predicted} \end{array}\; \end{array}\right.. \end{array}$$

The needle insertion distance $\hat{s}$ is linearly projected into the prediction horizon *N*. The thresholds *m*_1_ and *m*_2_ are experimentally determined (Narayan et al., 2018b).

### 4.2 Real-time prevention algorithm

**FIGURE 4:**
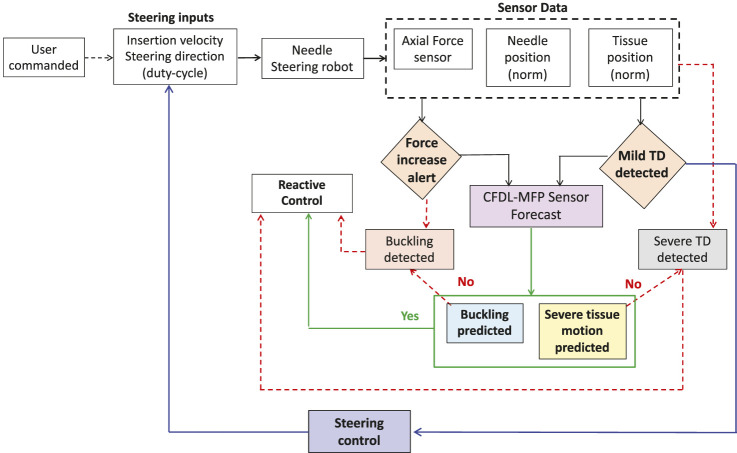
Flowchart for the overall prevention of adverse events. Critical events such as STD and needle buckling are prevented using reactive control (e.g., robot stop and retract needle). When mild events are detected, needle insertions continue through optimal steering control from IMFAC to prevent potential severe events before occurrence.

To prevent critical events such as needle buckling and STD before they occur, it is necessary for the robot to respond immediately to the prediction algorithms. For this purpose, reactive control is implemented where the robot stops and retracts the needle when buckling is predicted, and the robot stops insertion when STD is predicted to avoid potential damage to the tissue. However, when a mild event such as MTD is detected, adaptive control (IMFAC) computes optimal steering inputs (insertion velocity and duty cycle) to steer needles such that the occurrence of mild events is minimized and severe events are prevented. This strategy ensures continuity of the insertion procedure which otherwise is disrupted by reactive control. A complete event prevention algorithm with a combination of reactive and adaptive control is shown in Figure 4.

Algorithm 1 Adverse event prevention.

**Require:** Axial force sensor *f*_*a*_(*k*), 3D needle-tip sensor ${\vec{p}}_{nw}\left(k\right)$ , 3D tissue sensor ${\vec{p}}_{tw}\left(k\right)$ , prediction horizon *N*, total insertion distance *s*, commanded insertion velocity *ν*, and commanded duty-cycle factor *D*.
1:  *k* ← 0
2:  Initialize IMFAC parameters (*λ*_*c*_, Θ_*c*_(0), *u*_*c*_(0) = [*ν*,*D*]^*T*^, *l*_*tw*_(0))
3:  Initialize CFDL-MFP parameters
4:  PredictionFlag ← 0
5:  **while** *s*(*k*) < *s* **do**
6:  *l*(*k*) ← Norm $\left(\vec{p}\left(k\right)\right)$ ; $\vec{p}=\left({\vec{p}}_{nw},{\vec{p}}_{tw}\right)$

7: $f_{p}^{*}\left(k\right),l^{*}\left(k\right)\leftarrow$ *OptimalMean* $\left(f_{a}\left(k\right),l\left(k\right)\right)$ ; *l* = (*l*_*nw*_, *l*_*tw*_)
8:  *k*_*f*_, *k*_*b*_ ← *CheckBuckling* $\left(f_{a}\left(k\right),f_{p}^{*}\left(k\right),l_{nw}\right)$

9:  **if** *k* = *k*_*f*_ **then**
10:   Force increase alert
11: ${\hat{f}}_{aN}\left(k\right)=$ CFDL-MFP $\left(f_{a}\left(k\right),f_{p}^{*}\left(k\right),N\right)$

12:   PredictionFlag ← *CheckBuckling* $\left({\hat{f}}_{aN}\left(k\right),f_{p}^{*}\left(k\right),l_{nw}\right)$

13:  **end if**
14:  **if** PredictionFlag OR *k* = *k*_*b*_ **then**
15:   Buckling predicted/detected: Insertion stopped and needle retracted
16:  **end if**
17:  (*k*_*m*_, *k*_*s*_) ← *CheckTD* $\left(l_{tw}\left(k\right),l_{nw}\left(k\right),s\left(k\right)\right)$

18:  **if** *k* = *k*_*m*_ **then**
19:   MTD detected
20: ${\hat{l}}_{\mathrm{twN}}\left(k\right),{\hat{l}}_{\mathrm{nwN}}\left(k\right)=$ CFDL-MFP $\left(l_{tw}\left(k\right),l_{nw}\left(k\right),l_{tw}^{*}\left(k\right),l_{nw}^{*}\left(k\right),N\right)$

21:  PredictionFlag ← *CheckTD* $\left({\hat{l}}_{\mathrm{twN}}\left(k\right),{\hat{l}}_{\mathrm{nwN}}\left(k\right),\hat{s}\left(k\right)\right)$

22: **end if**
23: **if** PredictionFlag OR *k* = *k*_*s*_ **then**
24:   STD predicted/detected: Insertion stopped
25:  **else**
26: $\left(u_{c}\left(k\right),\Theta_{c}\left(k\right)\right)\leftarrow$ IMFAC $\left(l_{tw}\left(k\right),\lambda_{c},\Theta_{c}\left(k-1\right),u_{c}\left(k-1\right)\right)$

27:  **end if**
28:  *k* ← *k* + 1
29:  **end while**

The pseudo code for adverse event prevention is presented in Algorithm 1. The code is an integration of the proposed methods with the previous event detection, sensor forecasting (CFDL-MFP), and prediction algorithms (Narayan et al., 2018a; Narayan and Fey, 2020). The inputs to the algorithm are the filtered sensor data at current time *k*, CFDL-MFP prediction horizon *N*, user-commanded initial needle insertion velocity *ν*, and duty-cycle factor *D*. Before iteration, the IMFAC control parameters are initialized according to Section 3.2, and the initial event prediction flag is set to zero, see steps 3–4. The total insertion distance *s* is user-defined prior to the insertion procedure. Encoder readings *s*(*k*) are used to check if the measured insertion distance reached user-defined *s*, see step 5. All sensor data are filtered in real time using the Kalman filtering scheme from the work of Narayan et al. (2018b). For every iteration, the Euclidean norm of 3D position sensors and optimal mean forces $f_{p}^{*}\left(k\right)$ are computed, since CFDL-MFP and detection algorithms work on univariate time series data, see steps 6–7. The *Optimal Mean* function is the running average of the observed sensor data (Narayan et al., 2018a). Checks for needle buckling detection are performed for every *k*, see step 8. If a force increase is alerted by the detection algorithm, *N*-step forecasts of force data ${\hat{f}}_{aN}\left(k\right)$ are generated with CFDL-MFP and the buckling prediction algorithm checks for any buckling event using the force sensor forecasts (Narayan and Fey, 2020). If buckling is predicted, the robot stops insertions and needle is retracted, see steps 9–16. Similarly, checks for tissue displacement detection are performed, see step 17. If MTD is detected (*k* = *k*_*m*_), the displacement prediction algorithm checks for STD events. When STD is predicted, the robot stops insertions, see steps 18–24. Otherwise, steering control inputs for the needle are continuously updated until the end of insertion distance *s* to minimize or prevent further displacements of the tissue, see steps 25–29. The algorithm is executed in a multi-threaded framework to ensure simultaneous prediction and prevention of adverse events in real time.

## 5 Experimental methods

This section describes in detail the robot hardware setup and protocols used to validate the adverse event prevention algorithm during needle insertions in artificial and biological tissues.

### 5.1 Robot and setup

**FIGURE 5:**
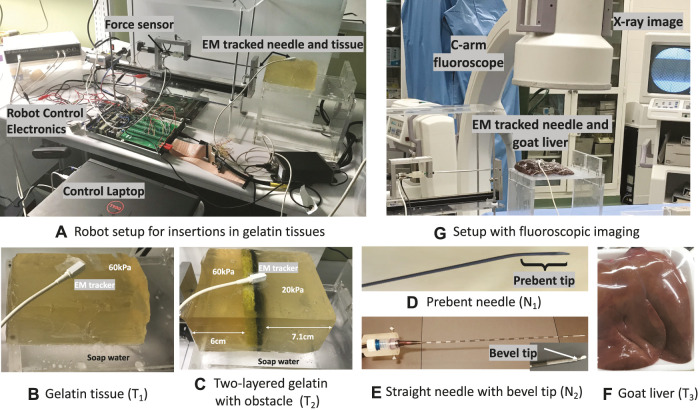
Experimental setup: **(A)** Needle steering robot with force and EM sensors. **(B,C)** Gelatin tissues (*T*_1_, *T*_2_) on a slippery platform smeared with soap water to elicit adverse events. **(D,E)** Prebent-tip needle (*N*_1_) for insertions in all tissues and the straight bevel needle (*N*_2_) for insertions in *T*_1_ and *T*_2_. **(F)** Goat liver (*T*_3_) on a non-slippery platform to validate event prevention algorithms. **(G)** Fluoroscopic imaging to track adverse events during insertions in *T*_3_.

The needle steering robot for needle insertion experiments is shown in Figure 5A. The robot is controlled with a Dell Inspiron 15 Gaming Laptop through custom C++ multi-threaded application (QT Creator 4.6.0) on Ubuntu Linux 16.04. The code manages low-level PID motor control and the execution of duty-cycled flipping algorithms to enable different needle steering directions (Majewicz et al., 2014). The robot is equipped with a 6-axis force–torque sensor (Nano-17, ATI Inc.) at the needle base to track axial forces due to needle–tissue interaction and an electromagnetic (EM) tracker (trakSTAR, NDI Inc.) to track needle-tip position. Data from these sensors are streamed at 50 Hz similar to (Narayan et al., 2018b). Algorithm 1 is coded as modules in Python 3.5 and interfaced with a low-level motor control code in QT Creator C++ through pybind11, an open-source Python-C++ wrapper that calls Python modules from C++ directly enabling multi-threaded operations. Real-time plotting of sensor data and prediction results of Algorithm 1 during needle insertions are displayed on QT GUI using the QCustomPlot library.

### 5.2 Gelatin tissues to simulate adverse events

A homogeneous gelatin tissue of stiffness 60 kPa (1:3 gelatin to sugar in 500 mL water) and dimensions 14 × 8 × 7.5 cm was created to validate tissue displacement prevention methods from Algorithm 1. The tissue is placed on a platform smeared with soap water to simulate a slippery surface for the sliding motion of the tissue, as shown in Figure 5B. For short-hand notation, let this sample be denoted as *T*_1_. Another gelatin sample of varying stiffness 60 and 20 kPa (1:3 gelatin to sugar in 1000 mL water) was created by embedding an obstacle to validate needle buckling prevention methods in heterogeneous tissue with a known environment. To prevent damage to the needle and the EM needle position sensor, a standard rectangular Scotch-Brite dish scrubber of thickness 0.7 cm is used as an obstacle. The 20 kPa region is on one side of the obstacle at 7.1 cm from a short edge, and the stiffer 60 kPa region is on the other side of the obstacle at 6 cm from the opposite short edge as shown in Figure 5C. This tissue denoted as *T*_2_ is also placed on the slippery platform to validate Algorithm 1 during buckling and tissue displacement events. For more details on gelatin preparation methods, refer to the work of Narayan et al. (2018a). Both *T*_1_ and *T*_2_ are used for experiments within 12 h of refrigeration. A trakSTAR sensor was taped on the surface of these tissues to track displacements.

### 5.3 Steerable needles

Two steerable needles with different dimensions and tips are used to validate the proposed prevention methods for robustness to needle types. The first steerable needle (*N*_1_) shown in Figures 5D is made from a hollow nitinol tube with dimensions 250 mm length, 0.8 mm OD, and 0.6 mm ID and a 30° prebent tip of 4 mm length (Majewicz et al., 2014). For integration with the robot, an EM needle tracker was inserted into the hollow end of the needle and secured with Luer locks. The second needle (*N*_2_) is a clinically available prostrate seeding needle with a stiff hollow tubing of length 175 mm and thickness 1.27 mm. A stainless steel wire of thickness 1.2 mm was cut into a small piece of length 10 mm and inserted into the tip of the hollow seeding tube such that 5 mm was jutting out of the tube and the rest 5 mm was inside the tube. The tip was ground to create a bevel angle of 60° to enable cutting through the tissue. Another EM needle tracker was inserted into the hollow end of the needle and secured with Luer locks for integration with the robot similar to *N*_1_, as shown in Figure 5E.

### 5.4 Insertion protocol in gelatin and biological tissues

To specifically evaluate IMFAC control and tissue displacement prevention methods against different needle types, a total of 30 insertions with prebent needle *N*_1_ and six insertions with straight needle *N*_2_ were performed in tissue *T*_1_. To validate both buckling prevention and tissue displacement prevention algorithms against various needle and tissue types, a total of 10 insertions with needle *N*_1_ and four insertions with *N*_2_ were performed in tissue *T*_2_. These insertions were performed using four initial user-commanded insertion velocities (0.25,0.5,1,1.5)cm/s, and three initial user-commanded duty cycle (0,50,100)%. Videos of the insertions were recorded using an iPhone 11 camera focused on the top surface of the tissue and a digital camera (Sony W800/B20MP 5X Optical Zoom) focused on the overall robot-tissue setup. The videos were used as ground truth for verification of the prevention algorithms. For viewing and analysis, motion images were generated in MATLAB at 30 frames per second and overlaid using Adobe Photoshop CC 2020.

Around 16 needle insertions were performed with the *ex vivo* goat liver tissue labeled as *T*_3_ in Figure 5F at the University of Texas Southwestern Medical Center. The liver tissue was placed on a clean platform. Live fluoroscopic videos were collected using a 9-in XRII OEC series 9600 fluoroscope (X-ray source and CCD camera) and saved with a Canopus ADVC 110 frame grabber, as shown in Figure 5G. The fluoroscopic videos were used as ground truth to check if adverse events were detected or prevented using Algorithm 1.

## 6 Results and discussion

**FIGURE 6:**
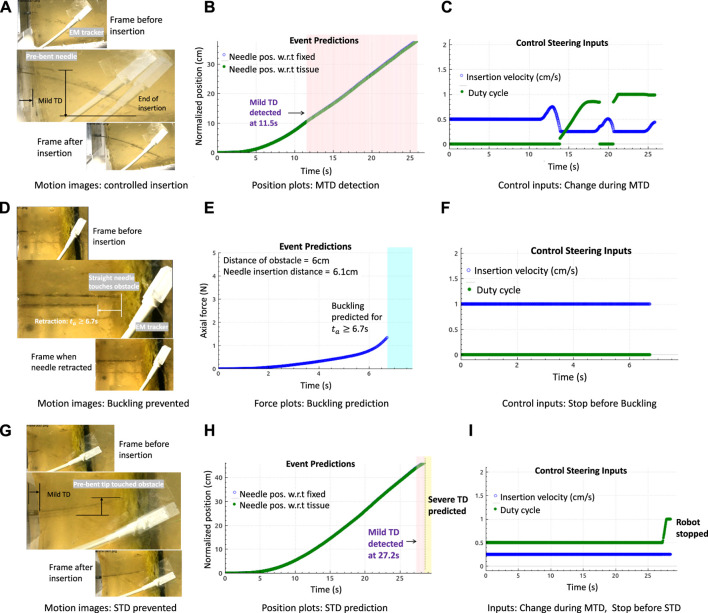
Results in gelatin tissues: **(A–C)** Motion image and plots showing controlled needle insertions with the prebent needle (*N*_1_) in tissue *T*_1_ to minimize tissue motion, by changing needle insertion velocity and duty cycle (tip orientations) during the detection of MTD. **(D–F)** Motion image and plots showing the retraction of the straight needle (*N*_2_) at the obstacle in *T*_2_, and buckling was predicted. **(G–I)** Motion image and plots showing controlled needle insertions with needle *N*_1_ in tissue *T*_2_ during MTD, followed by reactive control (robot stopped insertion) when severe displacement (STD) was predicted.

A good result of an optimal needle steering in the event of a mild tissue displacement is shown in Figures 6A–C. As the prebent needle *N*_1_ steered within the tissue *T*_1_, MTD was detected in the direction of the tip showing curved tissue motion from the motion image in Figure 6A. Each motion image was generated by overlaying three frames of motion above the other to show the translation displacement of the tissue and insertion or retraction motions of the needle. As soon as MTD was detected at 11.5 s (pink-shaded region) from Figure 6B, optimal steering inputs were calculated such that MTD stopped for short periods of time as can be seen in the discontinuities around 20 s from Figure 6C. The insertion procedure completed with reduced insertion velocity (blue plot) and increased duty-cycle (green plot) as no severe events were predicted. It should be noted that the insertion inputs were constrained where the velocity cannot reduce below 0.25 cm/s nor increase beyond 1.5 cm/s due to motor ratings. Similarly, there are only limited duty-cycled steering options between 0% and 100%. If the needle was inserted with the lowest insertion velocity and MTD was detected, then the duty-cycle increased to reduce tissue motion.

Buckling prediction results in tissues with embedded obstacle *T*_2_ are shown in Figures 6D–F. The motion images are obtained by overlaying two frames above the other to show the insertion and retraction motions of the needle during a predicted event. The frames corresponding to the interaction of the needle tip at the obstacle and the final retraction step are overlaid for better visibility since overlaying more than two images causes blur. In all the plots shown, buckling events were predicted at those timestamps when a force increase (not shown) was alerted by the detection algorithms as soon as the needle tip touched the obstacle, and the needle retracted as soon as a buckling was predicted at this time of force increase alert at 6.7 s. Using Algorithm 1, the robot stopped insertions and the needle was retracted by 2 cm. The retraction distance of 2 cm was chosen arbitrarily. This choice would depend on the path planning algorithms based on preoperative data or the operating surgeon. Results in the 20 kPa region of tissue *T*_2_ show that prediction algorithms are robust to tissue environments and that they solely depend only on the force readings. However, the limitation is that sometimes, force does not increase even on encounter with an obstacle. In this case, the algorithms would neither detect nor predict any buckling event, explained in the next paragraph.

An example of an optimal needle steering with reactive control is shown in Figures 6G–I. When *N*_1_ was inserted into tissue *T*_1_, MTD was detected at 27.2 s as soon as the tip approached the obstacle. Optimal steering inputs were calculated to reduce MTD, but the robot stopped motion as severe displacement of the tissue was predicted, represented by the yellow-shaded plot. It should be noted that buckling of the needle shaft was expected as soon as the tip encountered the obstacle; however, instead of a rapid force increase, a mild displacement was detected which resulted in severe displacement. Therefore, even when the needle appeared to have buckled, the axial force did not increase rapidly as expected, which caused both the buckling prediction and detection algorithms to fail. However, the overall procedure worked because of a displacement prediction and the insertion stopped on time. This example shows the advantage of developing unified methods for adverse event predictions.

**FIGURE 7:**
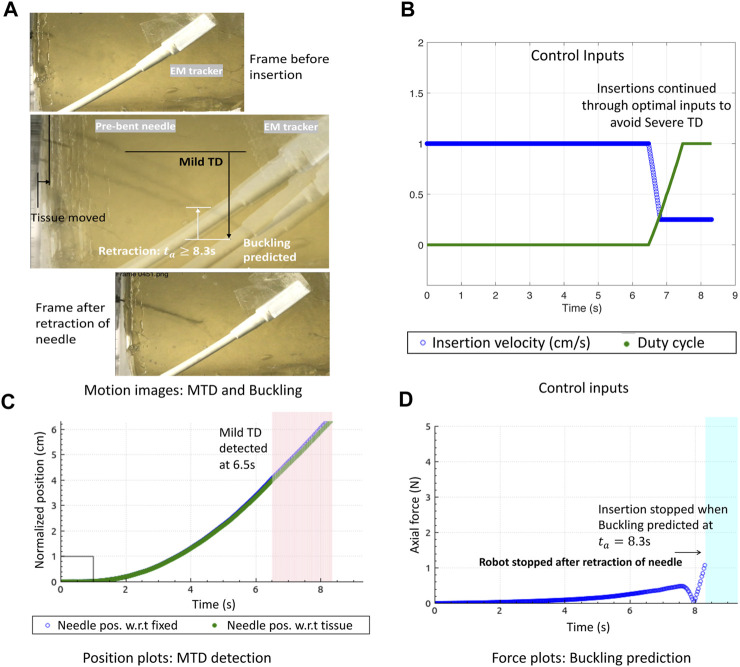
Optimal needle steering control with buckling prevention: **(A)** Motion image showing optimal needle insertions in gelatin *T*_1_ to minimize MTD and prevent needle buckling. **(B)** Control inputs change when MTD is detected to avoid STD. **(C)** Normalized position plots of the needle and tissue showing MTD detections. **(D)** Buckling predicted due to which the robot stops insertion and retracts the needle.

Another example of a unified prediction is shown in Figure 7. When the needle *N*_1_ was inserted for 0% duty cycle, the needle became curved due to the high stiffness of the tissue. Moreover, since the surface was slippery, mild displacements were detected as the needle steered further into the tissue. Due to the high steering curvature of the 0% duty-cycled needle, a buckling was predicted and a slight bend of the needle shaft can be seen in the motion image. Even though this could be a false buckling alarm, it is still safer to stop the needle as it gives time to the operating surgeon to decide if the insertion procedure should continue or stop.

**FIGURE 8:**
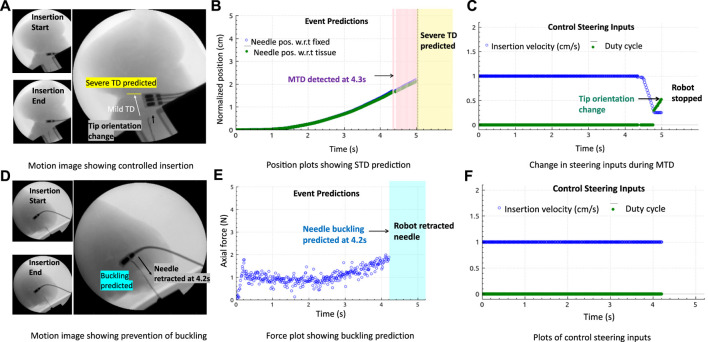
Prevention of adverse events in biological tissue: **(A–C)** Fluoroscopic motion image and plots showing controlled insertions with the change in insertion velocity and duty cycle (tip orientation) during MTD, followed by reactive control (robot stopped insertion) when STD was predicted. **(D–F)** Motion images and plots showing needle retraction when a drastic increase in force was predicted.

**FIGURE 9:**
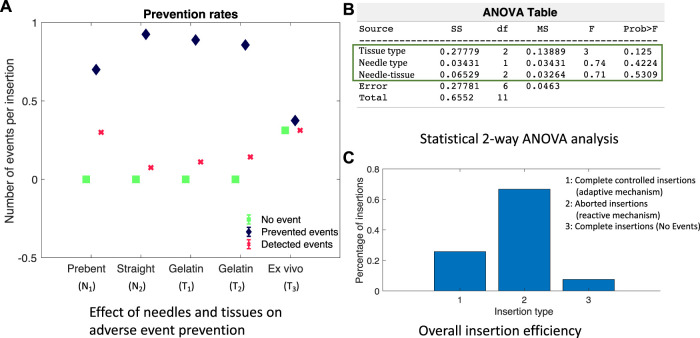
Performance of adverse event prevention algorithm: **(A)** Adverse event occurrence rates for different needles and tissue environments. **(B)** ANOVA analysis screenshot from MATLAB showing the effect of needle–tissue interactions. **(C)** Percentage of insertions controlled or aborted to prevent adverse events.

Validation results in the *ex vivo* goat liver *T*_3_ are shown in Figure 8. The prebent needle *N*_1_ was used for all insertions in *T*_3_. The timestamps of the live fluoroscopic videos match the timestamps of the real-time plots of the predicted adverse events, confirming the timely prevention of these events in heterogeneous tissues. All insertion data in gelatin and goat liver tissues with both the types of needles were combined for statistical analysis. The Kruskal–Wallis statistical method was performed on the data to study the effectiveness and robustness of the prevention algorithm against different needles and tissue environments. Prevention rate is computed as the ratio of the number of adverse events prevented to the total number of insertions for each tissue sample and needle type (blue diamond bars), see Figure 9A. Similarly, the detected event rate (red cross bars) is the ratio of the number of adverse events detected by the total number of insertions for each tissue sample and needle type, and the rate of no events is computed as the ratio of the number of insertions with no adverse events detected or predicted by the total number of insertions for each tissue sample and needle type (green square bars). Error bar plots show that the largest percentage of the needle insertions was optimized for control and prevention of adverse events (blue diamond bars). According to the two-way ANOVA analysis from Figure 9B, prevention rates did not depend on the needle or tissue type nor the needle-tissue interaction effects (*p* > 0.01), proving the robustness of the algorithms. The rate of detected events shows that early prediction of adverse events was not successful; however, the robot still stopped the insertion procedure through a reactive control mechanism on detection of the adverse event. The rate of controlled needle insertions (prevented events) in the goat liver was comparable to the rate of the detected events because of the local deformations of the tissue which were detected as severe tissue displacement events. Tissue deformation is a separate problem that is not covered in this work; however, real-time tissue mapping could be used to obtain control inputs for optimal needle steering with minimal tissue deformation and motion.

To study the effectiveness of the adverse event prevention algorithm to robotic needle steering applications, needle insertions were grouped into a) completed insertions with an optimal steering mechanism to prevent severe adverse events, b) aborted insertions due to the prediction or detection of a severe event, and c) completed insertions with no adverse events predicted or detected. The bar plots in Figure 9C show that the majority of needle insertions were aborted due to the reactive control mechanism of Algorithm 1 which prevented severe events such as STD and needle buckling. In about 25% of the needle insertions, MTD was detected and optimal steering inputs were continuously updated to complete the insertion procedure while minimizing MTD and preventing severe events. About less than 10% of the needle insertions did not encounter any adverse events, which shows that reactive and adaptive control mechanisms are important to be considered in robot-assisted needle procedures to ensure patient safety.

A major limitation of this study is that there were no targets assigned to evaluate the accuracy and time of the insertion procedure. Steering control inputs should be optimal not only in minimizing adverse events but also in procedural time; that is, time taken for the needle to reach desired targets, while also minimizing adverse events simultaneously, needs to be extensively studied and is a scope for future work. This work is a significant step to ensure procedural safety through data-driven techniques. When combined with efficient computer vision and tactile feedback techniques, our prediction and control methods can significantly improve patient safety and reduce procedural time and effort for the operating surgeon.

## 7 Conclusion and future work

We improved the existing model-free adaptive control technique (IMFAC) for general nonlinear systems to ensure continuous system stability and robustness against changes in system parameters and external disturbances. Comparing the simulation results with existing MFAC, IMFAC was more stable and robust to arbitrary values of system and control parameters. Then, we developed a preventive control subroutine by integrating IMFAC with previously developed event prediction methods from the work of Narayan and Fey (2020) to minimize adverse events in real time. Choice between reactive and adaptive control techniques was autonomous depending on the severity of the event. The robot implemented reactive methods such as needle retraction and stopped insertions when a needle buckling or a severe tissue displacement was predicted. During a mild tissue displacement, the robot adaptively steered the needle into the tissue to minimize and prevent future severe displacements so that continuity of the insertion procedure is ensured. Statistical results across different tissues and needles show robustness and generalizability of our algorithms to incision tool types and unknown tissue environments.

Since the algorithms rely on sensor data, the prediction and prevention subroutines might fail if a sensor gets faulty, resulting in more severe adverse events and injuries to the tissue. To address this issue, future work will focus on integrating augmented visual feedback techniques with our data-driven models. Moreover, the technique would also help identify the source of adverse events so that more intelligent actions can be implemented to ensure better continuity and safety of the procedure.

## Data Availability

The raw data supporting the conclusion of this article will be made available by the authors, without undue reservation.
